# Re-Thinking Gender, Artivism and Choices. Cultures of Equality Emerging From Urban Peripheries

**DOI:** 10.3389/fsoc.2021.637564

**Published:** 2021-03-09

**Authors:** Tatiana Moura, Linda Cerdeira

**Affiliations:** ^1^Centre for Social Studies, University of Coimbra, Coimbra, Portugal; ^2^ Centre for Social Studies, University of Coimbra, Coimbra, Portugal

**Keywords:** culture, peripheries, feminism, artivism, masculinities

## Abstract

Gender dimensions of violence, and especially women’s experiences in settings of urban violence have been the subject of important feminist research, including those that highlight gender as essential for comprehensive analyses of security and urban violence, and for promoting solutions and positive change. A primary contribution of feminist research indeed has been to demonstrate that there are both visible and invisible aspects of urban violence. A gap in literature on these gender dimensions is that of men’s construction of masculinities – and how these constructions are challenged during times. An important set of invisible phenomena within urban spaces and their peripheries includes the positive and decolonial responses that occur, including non-violent and feminist cultural and artistic pathways and the factors that lead men to resist to dominant, violent, or ‘hyper’ versions of masculinities. While there is a predominate focus on men’s involvement in violence, far less attention has been placed on men’s non-violent pathways. Based on examples of cultural, artistic and activist practices from the peripheries, namely those emerging in Rio de Janeiro, this article aims to discuss how artivism can challenge gender inequalities and power relations.

## Introduction

The mainstream debate on public security and urban violence in Latin America and elsewhere generally focuses on drug-related violence, kidnappings and violence between rival gangs. Considerable research has explored risk factors such as alcohol consumption, access and availability of guns, population density, poverty, unemployment, the lack of governance and the weakness of the state (Muggah, 2012). Yet, these analyses have often failed to fully explain the complex configuration and tragic persistence of violence in urban settings as well as the drivers of the inequitable gendered distribution of violent deaths, which are linked and reflect gendered, societal hierarchies and power relations in terms of how such violence disproportionately affects young, non-white and poor men in deeply polarized, unequal and segregated cities. The latest Brazilian Public Safety Yearbook (Cerqueira et al., 2019), for example, indicates that homicide victims in Brazil are mostly black (75.4%), between 18 and 29 years old (68.2%) and only studied through primary school (81.5%). Similar trends have been observed in other countries of the Americas (Hideg and Frate, 2019).

However, some gender dimensions of violence, and especially women’s experiences in settings of urban violence and war have been the subject of important feminist research, including those that highlight gender as essential for comprehensive analyses of security and urban violence, and for promoting solutions and positive change (Moser, 2004; Moura, 2007; Sjoberg and Via, 2010; Moura and Santos, 2011; Moura et al., 2012). This research has also drawn attention to and posed questions regarding interconnections between violence in public (including urban spaces), and intimate partner violence. A primary contribution of feminist research indeed has been to demonstrate that there are both visible and common effects of urban violence and wars: often what is registered or manifested in public, urban spaces; as well as invisible effects, those that remain ‘unaccounted for’ (see Barker, 2005; Moura, 2007; Moura et al., 2009; Moura and Roque, 2009; Roque, 2012a; Moura and Santos, 2012).

A major gap in literature on these gender dimensions is that of how boys are socialised into men – young men’s construction of masculinities – and how these constructions are challenged during the socialisation process. These constructions are met with expectations to provide for the family (with enhanced pressures in times of economic strain); and often, the option to use violence as a tool to achieve status, exert power or control that is threatened or lost, and to achieve social belonging. However, an important set of invisible phenomena in feminist analysis and masculinities studies include the positive responses that occur, including young men’s non-violent pathways and the factors that lead them to resist to or cope with dominant, violent, or ‘hyper’ versions of masculinities in urban contexts. While there is a predominant focus on men’s involvement in violence, and a tendency to position young men as a problematic group, far less attention has been placed on young men’s non-violent pathways (Barker, 1998; Barker, 2005; Roque, 2012b), namely how young men resist to or cope with dominant versions of masculinities. Understanding what leads men to prioritise non-violent pathways is key to preventing intergenerational transmissions of violence (Barker et al., 2012).

Based on examples of cultural and artistic practices from the peripheries, namely those emerging in Rio de Janeiro, this article aims to discuss how performance as an artivist practice allows one not only to denounce racist, sexist and colonial practices (past and present), but also to reflect on their potential for coalition around common struggles and guaranteeing the right to the city. It is intended to discuss the importance of the use of narratives told in the first person, in relation to the crystallization or emancipation of the performativity of bodies and the occupation of public space by subalternized bodies, challenging the cultural and social norms built around the possibilities of being.

Based on previous work conducted and published by the authors (Moura and Fernandez, 2020) it discusses how artistic and cultural practices in urban peripheries can transform relations of inequality, decolonize language and open space for hidden narratives in the peripheries. It argues that cultural practices such as dance, music and slam poetry performed by peripheral subjects contribute to a redefinition of identity building and sense of belonging to the city, taking into account intersecting oppressions of gender, race and class that informs daily life and positioning of particular subjects at not simply geographical peripheries, but at the edges of socio-economic structures and relations. Understanding the negative representations and stereotyping of urban peripheries is essential to the building of a new perspective away from the notion of “absence” and understand how they are sites of creativity and potentiality which can be seen through the production of arts and cultural practices that affirm rights and promote gender and racial equality, non-violent life trajectories and spaces of resistance.

## Masculinities, Non-Violent Pathways, Resistance and Coping

Multiple contemporary ‘crisis’ scenarios with intersecting layers of complexity and underlying causes emerge in urban spaces worldwide. These scenarios are marked by overlapping factors of financial and health crisis, histories of war, and violence in urban spaces – and the interplay between masculinities and violence in these settings – all of which pose complex and unresolved challenges.

The study of *newest wars* offers several starting points in order to consider the nature and effects of crises. “Newest wars” occur largely in scenarios of formal peace, cities and their peripheries, marked by socio-economic factors and disparities, and gender ideologies (Moura 2007; Moura 2010). A high concentration of state and gang violence within larger scenarios of institutionalised peace in these contexts stem from combined social and economic asymmetries, high unemployment rates, unplanned city growth, the availability of firearms and a centrality of violent norms, among other factors (Cockburn, 2001; Moser, 2001; Moura, 2007; Moura et al., 2009; Moura, 2010; Santos et al., 2011).

A major gap in the feminist literature about urban violence, peace and conflicts is how boys are socialised into men – young men’s construction of masculinities – and especially how these constructions are challenged during time.

Masculinity is the “set of norms, values and behavioural standards that express explicitly or implicitly expectations on how men should act and present themselves before others” (Miescher and Lindsay, 2003:4), in a gender order. They are complex, heterogeneous, part of a relational notion of gender in which they are not isolated from, but interact with femininities and diverse influences (Connell, 2005b), and they are fluid, dynamic and may change over time.

Most of the global studies on masculinities from the last 30 years shed some light on these aspects, with a special focus on the factors that contribute to male use of violence in the public sphere. Globally, we know that violence, whether in conflict or otherwise, is disproportionately committed by men against other men. At the global level, men are three to six times more likely than females to carry out homicide, and men of all ages represent 80 percent of homicide victims (Krause et al., 2011).

Literature on urbanization, poverty and violence has contemplated multiple risk factors (urbanization, city density, poverty, inequality, theories around youth bulges and unemployment among young males, conflict-related legacies, and governance failures) (Muggah, 2012) – but often omits consideration of the connections between masculinities and each of these factors and factors associated with non-violent options. So, what does it mean to apply a masculinities lens to violence and conflict analysis? First, we affirm that while the socioeconomic and racial dimensions of urban violence have received attention (Sampson and Wilson 1995; Mark and Fraser 2015), gender, and within that masculinities, has not garnered significant attention. “Gender-neutral” concepts of urban violence continue to prevail. As a result, with some notable exceptions (see Pearce 2006; Wilding, 2011; Baird 2012; Kern and Mullings 2013; Peake and Rieker 2013; Wilding and Pearson, 2013; Wilding, 2014), gender relations, gender power dimensions and gender norms and identities have rarely or only superficially been considered in urban violence scholarship. Other gender analyses of conflict, urban violence and war simply disaggregate data on mortality or participation in armed groups by sex and call it a gender analysis.

Our analysis is informed by diverse border epistemologies, gender and masculinities theorists – from Raewyn Connell’s work on hegemonic and subaltern/margin masculinities to Judith Butler’s work on gender as performative (and masculinities as performed and judged by other men), to the vast array of work by Michael Flood and others on men’s use of violence in the context of partnered relationships and the complex interplay of individual, childhood, and societal conditions that drive that violence.

There are strong scientific, policy and programme implications for researching and working with young men and choosing as epistemological focus their non-violent pathways (and positive peace solutions in these contexts).

Theoretical discussions of masculinities have shifted from a more singular ‘male sex role’ view to a conceptualisation of ‘multiple masculinities,’ including an emphasis on change (Connell 2005b). This emphasis on change sheds light on alternatives to dominant/hegemonic forms of masculinities, and explicit attention to non-violent pathways. Understanding what leads men to prioritise pathways to non-violent and peaceful versions of manhood are essential to achieving peace and reducing violence, in the public and private sphere, and promote cultures of equality.

## Artivism, the Dismantling of Patriarchy and Hegemonic Masculinities: Pathways towards Disruptive Language of Power Structures

Urban peripheries across the global south and north are stereotyped as sites of violence and criminality. Such stereotyping negates not only the root causes and factors that (re)produce violence and conflict in the peripheries, but also the multiplicity of positive examples that exist within each context (Moura and Fernandez, 2020). Favelas across the city of Rio de Janeiro are imagined as territories apart from the city, as the non-city, vis-à-vis the formal city (Pinheiro 2011). Rio de Janeiro is structured around profound socio-spatial inequality producing what Santos (2007) terms, an “abyss and abyssal lines”*.* Santos (2007:71) argues that the Universe “on this side of the line” only prevails to the degree that it exhausts the field of relevant reality: beyond the line, there can only be invisibility and inexistence – or, at least, inexistence of relevant or understandable forms of being. More recently, Santos and Meneses (2010), in his Epistemologies of the South, challenges the centrality of the hegemonic eurocentric framework, broadening the production and understanding of knowledge to include perspectives of artists and activists in the transformation of colonial epistemological dominance. In this article we adopt this definition of artivism (resulting from the fusion between artistic expressions and social and political activism), drawing on “border thinking” as an ‘epistemology from a subaltern perspective’ (Mignolo and Tlotanova, 2006).

Art produced in and by the periphery, while frequently not recognised as ‘art’, has the potential to destabilise the discourse constructed by actors who are economically, socially, and racially privileged. These movements operate nationally and therefore have a large-scale possibility to contribute to cultures of equality. Art, in this sense, can greatly contribute to dismantling the construction, widely disseminated by the media, of the peripheral subject as a disempowered victim or a potential criminal, and the peripheries as a locus of absence (Moura and Fernandez, 2020). Silva (2016) propose a new decolonial approach by using the “paradigm of potential” to interpret social practices found in the favela, one which values the inventiveness and plural aesthetic expressions affirmed by residents in relation to their own experience of urban life. The art produced by peripheral subjects has the potential to produce and give visibility to another periphery which affirms itself through its agency, inventiveness, and non-violet pathways. Art can thus contribute to ensuring that peripheral subjects treated as inexistent may assert themselves and interfere in dominant representations of gender, race, and class.

Passinho and Slam Poetry (analysed bin the following section) are two examples of a multitude of cultural manifestations from the periphery that demonstrate the creative potential of groups to produce innovative artistic and cultural movements. These movements have potential to challenge structures of inequality founded on patriarchy, racism, heteronormativity, and coloniality by promoting spaces of inclusion for plural alternatives, non-hegemonic ways of bringing forward discourses of equality, occupying spaces, and shifting boundaries and meanings across spaces and bodies.

In contrast to the reproduction of models of masculinities based on the control and use of violence, Passinho and Slam resist the use of violence. These movements specifically call out state-backed violence and challenge the criminalisation of peripheral bodies. They refuse the stereotyping models of peripheral and black masculinities and highlight the complex struggle to exist in the imaginary of the police and society outside of oppressive structural inequalities. Slam poets are creating spaces, occupying city spaces, challenging intersecting inequalities, and promoting new understandings of the multiplicity of gender relations.

### Sewing the Broken City: Gender and Cultures of Equality

Previous work by the authors and other activists and researchers (Moura and Fernandez, 2020) state that cultural and artistic movements from the urban periphery in Rio de Janeiro can be seen within a perspective of equality. Many cultural movements promote “cultures of equality” by articulating demands at multiple levels: through verbal, physical, rhythmic, and/or aesthetic mediums; by creating equitable practices within their organisations; and by occupying and redefining spaces throughout the city. These movements promote spaces of inclusion for plural alternatives, non-hegemonic ways of bringing forward discourses of equality, and shifting boundaries and meanings across spaces and bodies. These were the basic assumptions of the GlobalGRACE project^1^. Underpinning this project were two basic ideas. The first was that equality is a cultural artefact and are made and contested in a variety of ways in different parts of the world. The second was that cultures might best be understood as the practices through which people create the worlds they inhabit, and people’s creative practices challenge inequality and engender new possibilities for more equitable ways of living together. In other words, GlobalGRACE treated gender equality as a contingent cultural product and that methodologically brought together interdisciplinary work to investigate the production and meanings of cultures of equality across a range of sites, events, practices and objects. Moreover, we adopted a critical de-colonial and postcolonial perspective that challenges the assumption that cultures of equality originate in and flow from countries in the global North. Our emphasis was on how new and alternative cultures of equality emerge from the periphery and out of situations of marginality. As Audre Lorde (2007) states:

The difference should not be merely tolerated but seen as a background of necessary polarities between which our creativity can sparkle as dialectics. Only then does the need for interdependence become non-threatening. Only within this interdependence of different, recognized and equal forces can the power to seek new ways of being in the world generate the courage and sustenance to act where there are no licenses.

This notion of interdependence of forces (and vulnerabilities) fosters not only a critical and conscious awareness of ourselves, but also of our body in relation to the outside - other bodies, structures, politics - besides allowing us to assume different roles according to the plurality of struggle scenarios.

In this sense we can affirm that artistic and cultural movements that arise naturally out of peripheries are creating spaces, occupying cities, challenging intersecting inequalities, and promoting new understandings of the multiplicity gendered identities – challenging gendered performativity and transforming vulnerabilities in collective forms of resistance. Given the connection of dominant versions of masculinity to the use of violence, it is shown that art performed in peripheral spaces can contribute especially to the production of non-violent and solidary forms of social organization.

This model that Mohanty (2003) calls a feminist model of solidarity focused on common interests, challenges contemporary feminist movements to reformulate their starting questions regarding what unites them and what separates them, and consciously perceive the interdependencies of their vulnerabilities and privileges, so that it is possible to develop strategies of struggle and resistance capable of transcending borders (geographical and/or cultural).

This model also values the narratives of historical experiences - collective and individual - challenging us to a permanent theorization and debate of the complexities and connections between the different experiences of being a man or a being a woman, democratizing instead of colonizing experiences. (Mohanty, 2003). The importance of understanding how identities and experiences change and flow over time becomes fundamental to the very process of resignifying struggles and coalition strategies.

People build identities that unite or separate them at these crossroads, often because of the exclusions or oppressions they are subject to and the forms they take. Although, these identities are neither fixed nor stable, they are not essential, but are subject to constantly changing contexts, and are therefore procedural, dynamic, unstable and tense, determined by contradictory logics of defining identities and differences. (Martins, 2018: 5).

Art and artistic performativity, in its multiple languages, as a device of social transformation, acts as a counter-hegemonic narrative before the production of hegemonic knowledge that dictates standardized behaviors and attitudes. The paths presented by the politically implicated artistic doing corroborate for a narrative that produces a theoretical-practical field with more possibilities, with processes of experimentation, projections and collective construction.

The performance, whether individual or collective, has the double role of not only exposing and denouncing violence, but also of democratizing its possibility of insurgency: what is said by the performance, whether by verbal language, or by the performativity of each body that stages it, ends up assuming a collective body, understood and assimilated by a plurality of individuals, regardless of having already suffered physical violence or not, regardless of their contexts, inside and outside their personal and collective histories. (Butler, 2018).

The artivist movements of Passinho and Slam Poetry are examples of how art creation and processes of production can challenge stereotyped notions of identity, promoting cultures that aim to reach more equal relations. Occupying spaces throughout the periphery and beyond, these movements are building networks that renegotiate the city’s divisions. While Slam can be seen to actively and overtly advocate for equality, challenging hegemonic power structures, Passinho inserts its participants into market forces, mainstream cultural movements, and showcases the value of individual dancers’ and groups’ talents. In both instances, essentialized images of the periphery and the criminalization of bodies and culture from these areas are challenged.

In fact, artivism as a political-aesthetic process was strongly influenced by the use of performance by artists linked to the feminist and queer movements (Mesías-Lema, 2018). Although the use of feminist performance has a long history from the 'second wave of feminism', its visibility has been largely attributed to western, white women artists, in line with the consolidation of the movement itself. As soon as the phrase 'the personal is political' was coined, women performers and artists began to turn their own experiences, using these as primary resources (Heddon, 2006: 130). The use of personal experiences that gave motto to the slogan 'The Personal is Political' was the strategy found to break with the invisibility of the so-called private sphere and oppressions. As hooksbell (2000) have long argued, telling the story in the first person allows the oppressed to become the subject of their own history, challenging their own power structures. In this sense, the narratives of the women who participate in slam poetry, particularly black and peripheral women, allow them not only to challenge the oppressive context, but also to emancipate themselves from their own experiences, so often marked by processes of violence. For this reason, after three decades of using so-called biographical performances, the reflection around their use as a tool of denunciation and resistance in the context of contemporary feminist struggles deserves to be re-examined, similar to the current challenges faced by the movements themselves.

Inspired by the *rap* and *hip-hop* movements of the 1970s, *slam poetry* as an artistic and performative language emerged in the United States in the 1980s. Mobilized by the poet Marc Smith in Chicago, it gained popularity in the following decades, particularly in the peripheries of urban environments not only in the United States, but also proliferating in various countries around the world (Freitas, 2020: 2).

In the particular case of Brazil, although *slam poetry* only consolidated in the 2000s, *hip-hop* as an urban culture gained strength in Rio de Janeiro in the 1990s, taking advantage of the space moved by the independent samba and choro circuits that began to take over the streets and alternative spaces in the city. Thus, no longer conditioned to ghettos and peripheries, *hip-hop* and the battles of spoken poetry began to consolidate and attract more and more public in the following years.

Notable groups and movements of slam poetry in Brazil include, *Slam Laje, Nós da Rua, Slam das Minas,* and *Slam Resistência*. *Slam das Minas*, in contrast with the other groups, is solely composed of women. This collective was first formed in 2015 in Brasilia and has grown quickly, expanding to other cities, notably Sao Paulo, Rio de Janeiro, and Porto Alegre. According to *Slam das Minas-Rio de Janeiro*, Slam das Minas is a playful poetic game to develop the artistic power of women. emerges as a feminist movement of marginal poetry in which women conquer their space: public space, artistic space and space to speak out and denounce their restlessness, violence and oppression. In their rhymes these women speak of love, of the power of the periphery, of patriarchal violence, of feminicide, of maternity, of pain and the power of being women, to multiple voices and multiple narratives. Slam das Minas, in addition to following the basic rules of *slam poetry,* modeled the format of their encounters with their own characteristics, thus acting strategically to challenge the structures of power.

Slam movements in Rio de Janeiro actively speak out against machismo, sexism, racism, homophobia, discrimination, violence, and other topics affecting the city’s residents. These movements articulate feminist agendas and anti-racism agendas defined by black women and people from the periphery both through individual poets’ contributions and organisation’s communications (Moura and Fernandez, 2020).

*Passinho* mixes different elements such as break, capoeira, kuduro, contortionism, mime, frevo, performance, combined with step marking in two counts, characteristic of funk carioca, electronic genre of track, created in the peripheries and communities of the city in the years 1980. In this way, Passinho imposes itself as a powerful expressive manifestation to reflect on new modalities of identity belonging in the second decade of 2000. It aligns with the debates on globalization, youth and new meanings of relationship between local and global, center and periphery, virtual and non-virtual (Bacal and Domingos, 2020). The dancers’ creativity and originality are key. Since 2008, Passinho *battles* have occupied numerous favelas, city metro stations, and the wealthier neighbourhoods in the *zona sul* (south) of Rio. Passinho gained global fame after being featured in the 2012 Olympic closing ceremony in London and the 2016 Olympic opening ceremony in Rio de Janeiro. In 2017, Passinho was declared cultural heritage under law 390/2017 (Rio de Janeiro 2017).

Under the scope of our project we partnered with Cia. Passinho Carioca, from Rio de Janeiro. Together, and in partnership with other movements and organizations, we asked: how does the art produced in and by the peripheries build subjectivities that rearrange and subvert everyday discourses and practices of hegemonic, white, and cisheteronormal masculinity? How do artistic and cultural interventions disturb the intersecting regime of gender inequality that structures us and disproportionately affects/oppresses the black and peripheralized population? How do they challenge stereotyped constructions about violent peripheralized masculinities? The final result was the show *Na Manha*, where the dancers are, through their performances, transgressing the gender boundaries internalized even in the Universe of Passinho; they are challenging the geographic and symbolic boundaries of a city that says how and where these bodies should transit and how they should perform; they are questioning the classist and racist limits of the city. In short, by freely performing with a gender and masculinities lens this young group of dancers in dialogue with two young female directors, black and of popular origin, are demanding another project of life. In this process, dancers resist the places and roles socially and violently constructed for their bodies to inhabit. Bodies crossed by markers of gender, sexuality, race, class and territory, which have been historically criminalized and dehumanized. Black men who have been socialized, as hooksbell (2018) teaches us, in a racist and macho culture that does not allow them to feel, to expose their vulnerabilities and of which a violent masculinity is demanded, can through art express a complexity of emotions. If the dominant culture has produced a culture of death of young men from the peripheries through secular processes of genocide, in the collective project *Na Manha* these bodies are made available for a culture of their own, centred on the affirmation of life.

We can recognize that these artistic and cultural practices take on a decolonial character that allows, even if momentarily, to break with normative expectations regarding the performativity of their bodies in a society deeply hierarchized by a social-spatial inequality.

## Discussion

Through this article we tried to analyse the implications that emerge from applying lens of power, patriarchy and masculinities to violence-affected areas, namely urban peripheries. From a gender and power analysis of peripheries, and adopting a feminist approach, we can go beyond the obvious, challenge stereotypes, and identify positive, creative and non-violent identities and practices. It is important to 1) understand salient power structures and gender norms in context, given the strong associations between masculinities, power, patriarchy and violence, in order to challenge directly gender and masculine norms in violence prevention efforts; 2) focus and identify resistance to patriarchal violence, for example by examining men´s resistance and resilience within contexts of violence, and nonviolent trajectories specifically, taking into account that “violent” or “nonviolent” do not comprise fixed categories. In addition, while the feminist literature increasingly focuses on men’s multiple experiences and expressions of gender, there remains a tendency to emphasize men’s involvement in violence rather than understanding their non-involvement, namely how men resist to or cope with dominant versions of masculinities. Risk and vulnerability, rather than resilience, is often a starting point for research on violence (amongst men). It is crucial to understand how to negotiate models of masculinity away from violence and how to produce alternative models of masculinity that resist violence and promote equality.

As previously mentioned, men’s violence is produced – not innate – and nonviolent, pro-social masculinities exist alongside violent versions of manhood in any setting, including in conflict settings. Understanding masculinities is never, on its own, the way to end conflict, and it should be done together with understanding women’s lived realities. It is by understanding patriarchy and masculinities, together with the array of other drivers of conflict and violence, and together with the experiences of women and girls, that solutions are likely to be more effective and more enduring.

This article politicises and questions the naturalisation of violence and multiple forms of oppression (gender, race, class, and geographical) that affect the favelas and favela residents. These forms of oppression cannot be explained without contemplating both past and present systematic inequalities. The peripheries in both the global South and the North, and in particular those of Rio de Janeiro, are presented here from the paradigm of potential, as spaces of creative agency. These spaces create movements that, on a daily basis, subvert power relations. Very briefly, we sought to understand artistic and cultural expressions that arise naturally out of peripheries and are creating spaces, occupying cities, challenging intersecting inequalities, and promoting new understandings of the multiplicity of gender identities – specifically peripheral and black masculinities. Given the connection of dominant versions of masculinity to the use of violence, it is shown that art performed in peripheral spaces can contribute especially to the production of non-violent masculinities.

In the contemporary world, permeated by so many complexities of existences, analyses and uncertainties, the common search seems to be in fact to find these possibilities of life: not only the guarantee that our bodies really remain alive, but that they are respected and not violated, that they circulate without fear, that they are accepted, that they move freely not only in physical territories but also in social structures and power structures.

## Footnotes

1 Global Gender and Cultures of Equality (GlobalGRACE) was a 51 months programme of research and capacity strengthening funded by the UKRI's Global Challenge Research Fund (GCRF) delivered through the Arts and Humanities Research Council. GlobalGRACE employed arts based practices and multi-sensory research to investigate the production of cultures of equality and enable gender positive approaches to wellbeing internationally. For further information, please consult https://www.globalgrace.net/
